# A High-Resolution SAR Focusing Experiment Based on GF-3 Staring Data

**DOI:** 10.3390/s18040943

**Published:** 2018-03-22

**Authors:** Mingyang Shang, Bing Han, Chibiao Ding, Jili Sun, Tao Zhang, Lijia Huang, Dadi Meng

**Affiliations:** 1 Key Laboratory of Technology in Geo-Spatial Information Processing and Application Systems, Institute of Electronics, Chinese Academy of Sciences, Beijing 100190, China; shangmingyang16@mails.ucas.edu.cn (M.S.); cbding@mail.ie.ac.cn (C.D.); zhangtao12@mails.ucas.ac.cn (T.Z.); ljhuang@mail.ie.ac.cn (L.H.); mengdadi@hotmail.com (D.M.); 2 Institute of Electronics, Chinese Academy of Sciences, Beijing 100190, China; jlsun@mail.ie.ac.cn; 3 School of Electronic, Electrical and Communication Engineering, University of Chinese Academy of Sciences, Beijing 100049, China; 4 National Key Laboratory of Microwave Imaging Technology, Institute of Electronics, Chinese Academy of Sciences, Beijing 100190, China

**Keywords:** SAR, GF-3, staring spotlight, two-step algorithm, curved orbit, stop-and-go, antenna pattern, high-resolution

## Abstract

Spotlight synthetic aperture radar (SAR) is a proven technique, which can provide high-resolution images as compared to those produced by traditional stripmap SAR. This paper addresses a high-resolution SAR focusing experiment based on Gaofen-3 satellite (GF-3) staring data with about 55 cm azimuth resolution and 240 MHz range bandwidth. In staring spotlight (ST) mode, the antenna always illuminates the same scene on the ground, which can extend the synthetic aperture. Based on a two-step processing algorithm, some special aspects such as curved-orbit model error correction, stop-and-go correction, and antenna pattern demodulation must be considered in image focusing. We provide detailed descriptions of all these aspects and put forward corresponding solutions. Using these suggested methods directly in an imaging module without any modification for other data processing software can make the most of the existing ground data processor. Finally, actual data acquired in GF-3 ST mode is used to validate these methodologies, and a well-focused, high-resolution image is obtained as a result of this focusing experiment.

## 1. Introduction

Synthetic aperture radar (SAR) has developed rapidly over the past few decades as an effective means of Earth observation. The resolution of spaceborne SAR has evolved from tens of meters to meters to even decimeters. Sliding spotlight (SL) mode in which an antenna always aims at a point called the virtual rotation point (VRP), allows for a longer accumulating time [1,2] and results in high-resolution images. Compared with the traditional stripmap mode, in which the VRP moves toward infinity, staring spotlight (ST) mode is another version of SL mode in which the VRP is just within the imaged scene on the ground [3,4,5,6]. Considering the multiple observation angles of SL/ST mode, Munson et al. treated SAR image focusing as a tomographic reconstruction problem of narrow-band computer-aided tomography (CAT) and proposed a tomography formulation [7]. Eichel et al. used spotlight SAR interferometry for terrain elevation mapping and interferometric change detection [8]. Based on the COSMO-SkyMed Spotlight-2 data, Filippo recovered partially corrupted SAR images by spectrum extrapolation [9]. In contrast to stripmap mode, both SL and ST modes have a wider Doppler bandwidth that is usually much larger than the pulse repeat frequency (PRF), which causes a Doppler aliasing phenomenon to occur in azimuth. One way to overcome this barrier to divide the azimuth echo data into small blocks that have a Doppler bandwidth that is smaller than the PRF, and a traditional stripmap mode imaging algorithm, such as the chirp scaling algorithm (CSA) [10] or the range Doppler algorithm (RDA) [11], handles each piece of data so that the processed blocks can be combined into a complete image [12,13]. Another method is the two-step processing approach proposed by Lanari [2]. The key point of this approach is a filtering operation in azimuth, which implements a convolution between the raw data and a chirp signal whose rate is selected—this step is also called azimuth prefiltering. Then, a classical stripmap SAR imaging algorithm can focus the prefiltered data into a SAR image. 

In reality, most SAR imaging algorithms are based on the acquisition geometry of airborne SAR. But the same approach used in airborne SAR and this experiment could not achieve the same performance, and there are two main reasons for this. Firstly, the acquisition geometry of airborne SAR can be depicted by the hyperbolic range equivalent model (HREM) [14], which is only able to describe the first- and second-order items of the range history. However, the third- and higher-order items would introduce significant phase errors in the signal processing when the azimuth resolution is in decimeter level. To solve this problem, Huang et al. added a linear term to the HREM to improve the model accuracy to the third-order, and the method for L-band SAR systems at altitudes from 1000 to 10,000 km could get an azimuth resolution around 3 m [15]. Having considered the effect of the Earth’s rotation, Eldhuset introduced a fourth-order model and derived the fourth-order extended exact transfer function (EETF4) algorithm to get better SAR images [16,17,18]. Wang et al. proposed a modified equivalent squint range model (MESRM), which introduces the equivalent acceleration of the platform into the HREM [14]. Henrion et al. described the sliding spotlight SAR imaging geometry by an Nth-order model [19]. But the complexity of the algorithm became more difficult as the order increased. Secondly, all of these approaches did not consider that the actual range history of the target varies with the target’s position. In other words, different targets in the imaged scene have different range histories. Based on the theory of motion compensation for airborne SAR, Han et al. made use of the range error caused by the non-linear movement of the spaceborne SL SAR relative to the VRP and corrected this kind of variance with a cubic item [20]. But in spaceborne ST mode, the VRP is just within the imaged scene and the approach in [20] could not compensate for the cubic phase error thoroughly. In this experiment, the range history of the reference target in the middle of imaged scene is calculated and the derivation between the actual and perfect hyperbolic range history can be obtained. We subtly compensate the derivation in azimuth-time domain. Then, the cubic phase error of every pixel in azimuth is calculated and compensated in range-Doppler domain.

Gaofen-3 (GF-3), a remote sensing satellite of China’s Gaofen project, which is the first C-band multi-polarization spaceborne SAR in China, was launched in 2016. GF-3 can work in 12 different modes, and some of its parameters such as incidence angle, nominal resolution, nominal swath, and polarization can be found in Table 1 in [21]. This satellite not only plays an important role in the fields of ocean surveillance, disaster reduction, and water conservancy, but also provides high-quality data for scientific experiments, which greatly promotes the development of SAR. In normal SL mode, GF-3 sets its antenna beam on a VRP below the ground while it flies in space, which can result in a trade-off between resolution and coverage in azimuth, and achieves the nominal resolution of 1 m with a swath of 10 km in both range and azimuth. In order to further explore GF-3’s potential in improving azimuth resolution, ST imaging experiments were performed after the satellite had been in operational application. In this mode, the antenna can always point to the same scene and get the highest theoretical resolution of about 42 cm by setting its steering angle range within ±1.9°. 

However, ST mode is not a regular imaging mode as shown in Table 1 in [21], and the original data processor cannot give qualified products. So there are still many difficulties that must be resolved before obtaining a well-focused image [22]. On the one hand, the stop-and-go approximation in imaging algorithms would be a bad influence on the quality of the image. This problem could be solved by introducing two items respectively in range-Doppler domain and two-dimensional frequency domain [23,24]. On other hand, GF-3 uses an active phased array antenna, but the steering angle range and power gain are modulated by array elements. This problem is very common in SL/ST and terrain observation with progressive scan (TOPS) [25] modes and can be solved by compensating the gain-loss ratio in the raw data level [26].

This paper introduces a GF-3 ST focusing experiment that aims to address problems in higher-resolution SAR images and make preparations for China’s next generation of spaceborne SAR. In Section 2, the data acquisition and preprocessing are illustrated briefly, and then, data processing including model error compensation, the imaging algorithm, stop-and-go approximation, and antenna pattern modulation is explained in detail. Experimental results are presented in Section 3, followed by discussion and conclusions in Section 4. 

## 2. Focusing Experiment Based on GF-3 Staring Data

**Figure 1:**
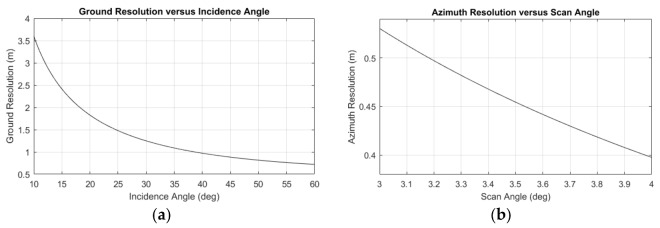
The resolution in ST mode. (**a**) The ground resolution versus the incidence angle; (**b**) The azimuth resolution versus the scan angle range.

In practice, to acquire operational SL-mode products, GF-3 works in the sliding spotlight mode during which the antenna steers at a VRP below the ground to increase the integration time. The significant distinction between SL mode and ST mode in this experiment is that in ST mode the VRP is located within the imaged scene on the ground (i.e., the antenna is always pointing at the same scene during the accumulating time). Table 1 summarizes some parameters of GF-3 in SL and ST modes. The resolution in slant range can be derived from the bandwidth of the pulse signal, but its projection on the ground—ground resolution—changes with the incidence angle as shown in Figure 1a. As we can see, GF-3 can get a ground resolution range within [0.815, 1.826] m. The resolution in azimuth changes with the antenna steering angle range as shown in Figure 1b. As we can see, GF-3 can get the highest theoretical azimuth resolution of about 42 cm, with a rectangular weighting Doppler spectrum, when the steering angle range is 3.8°.

However, higher resolution means more difficult processing, and the performance of traditional approaches usually is not satisfactory. In order to get qualified images, this experiment made some improvements to the range history model, the imaging algorithm, the stop-and-go approximation, and the antenna modulation. The details of this experiment can be found in the below subsections.

### 2.1. Data Acquisition and Preprocessing

GF-3 uses an active phased array antenna to achieve azimuth beam steering, which can steer faster compared to traditional mechanical antenna. In [21], Sun et al. introduced GF-3’s active phased array antenna, which contains 12 columns (azimuth) and 64 rows (elevation) that have transmit-receive channels measuring 7.5 m (azimuth) and 1.232 m (elevation) that are distributed in two panels in SL/ST mode. In consideration of its grating lobes (in Section 3.4), this antenna can achieve a steering angle within ±1.9°. At the same time, the steering angle varies discretely but continuously, and the step steering angle is 0.01°. In other words, the steering angle varies 0.01° after many pulses, which we call the stationary number of pulses (SNP). In effect, the SNP controls the VRP’s position when the PRF is constant; the larger the SNP, the farther the VRP is from the satellite. In this focusing experiment, the data was acquired on 11 March 2017 in Nanjing, China. The SNP is 90 and the SNP of a SL acquisition at the same scene is 120.

After receiving echoes reflected from ground, the processor on GF-3 compresses the data with a block adaptive quantization (BAQ) (8:4) algorithm. Every frame of the compressed data contains not only an echo, but also some essential auxiliary parameters, such as the radar system parameters, the satellite attitude and position, the antenna steering angle, and the beam position. 

Because the VRP is fickle due to the SNP, some tricks may be needed to determine the VRP’s position. We combine two frames of raw data into one equation and calculate the VPR. Assuming N frames of raw data are acquired during the integration time, there will be N/2 equations (i.e., the number of the VRP is N/2). Then, the final VRP is estimated by the least squares (LS) means. Moreover, some essential parameters like scene size, Doppler bandwidth (the whole scene and single point), and nominal resolution can be calculated.

At this point, both echo data and some essential auxiliary parameters have been acquired.

### 2.2. Curved Orbit and Cubic Phase Error

**Figure 2:**
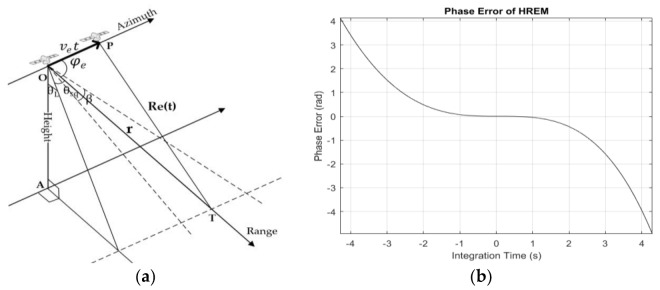
Schemes of hyperbolic range equivalent model (HREM): (**a**) The geometric figure of the HREM; (**b**) The model error between the actual range history and the equivalent range history in the ST experiment.

Initially, the traditional imaging algorithms were based on airborne SAR acquisition geometry that assumed that the target on the ground was still and the platform flew at a constant velocity. But in spaceborne cases, the platform’s flight path as well as the Earth’s surface are curved, and the Earth rotates continuously. In order to make the most of existing algorithms, the HREM, which used equivalent velocity and squint angle to describe the range history was proposed. This model (see Figure 2a) assumes that the sensor flies at a constant velocity $v_{e}$, the sensor locates on point O when the antenna points to the target T on the ground, and the azimuth time t=0. The sensor locates on point P at an arbitrary azimuth time t, A is the nadir point, the distance between O and T is r, the distance between P and T is $R_{e}\left(t\right)$, $\theta_{L}$ is the looking angle, $\theta_{sq}$ is the squint angle, and $\varphi_{e}$ is the complement angle of $\theta_{sq}$. Then, the range history can be expressed as:

(1) $$R_{e}\left(t\right)=\sqrt{r^{2}+{\left(v_{e}t\right)}^{2}-2rv_{e}t\cdot \cos\varphi_{e}}$$

(2) $$\{\begin{array}{c} v_{e}=\sqrt{\frac{\lambda rf_{R}}{2}+{\left(\frac{\lambda f_{D}}{2}\right)}^{2}}\\ \varphi_{e}=\arccos\left(-\frac{\lambda f_{D}}{2v_{e}}\right) \end{array}$$

where λ is the carrier wavelength; $f_{D}$ is the Doppler centroid frequency, and $f_{R}$ is the Doppler frequency modulation rate, and they can be obtained by calculating the first- and second-order derivative of the actual range history between the target and the sensor. In this experiment, some of these parameters are sketched in Table 2. 

In most cases, the integration time is usually short (no more than 2 s), and the trajectory can be described by the HREM accurately. In SL or ST mode, however, targets on the ground that are illuminated by a complete beam have a longer integration time as compared to stripmap mode. As shown in Figure 2b, the integration time of the target in the middle of the imaged scene is about 8.58 s. Because of the long integration time and the spaceborne acquisition geometry, the actual range history is not a pure hyperbola and the phased error caused by the range deviation becomes larger as the integration time extends. In addition, the third- and higher-order items of Equation (1) cannot be ignored in the long integration time or high-resolution. In operational cases, with the help of satellite attitude and orbit parameters provided by Global Positioning System (GPS), $f_{D}$ and $f_{R}$ can be calculated. Then, the equivalent range history can be obtained according to Equations (1) and (2), and we compensate the model error in two aspects—range deviation and cubic phase error.

In order to use the HREM to deal with raw data, the deviation between the actual and the equivalent range history must be compensated. The range history vector $R_{st}\left(t\right)$ of a target can be obtained with the help of attitude and orbit parameters, and it can be expanded by the Taylor formula:

(3) $$R_{st}\left(t\right)=R_{st}+V_{st}t+\frac{1}{2}A_{st}t^{2}+\frac{1}{6}B_{st}t^{3}+\frac{1}{24}C_{st}t^{4}+\cdots$$

where the subscript s means satellite, the subscript t means target; $R_{st}$, $V_{st}$, $A_{st}$, $B_{st}$, $C_{st}$ denote the distance, velocity, acceleration, rate of acceleration, and rate of rate of acceleration vector between the satellite and the target when t=0. Then, the distance between the satellite and the target can be expressed as

(4) $$R_{st}\left(t\right)=\left|R_{st}\left(t\right)\right|=r_{st}\cdot \sqrt{1+\frac{R_{st}\left(t\right)R_{st}\left(t\right)-r_{st}^{2}}{r_{st}^{2}}}=r_{st}+c_{1}t+c_{2}t^{2}+c_{3}t^{3}+c_{4}t^{4}+\cdots$$

where

(5) $$r_{st}=\left|R_{st}\right|$$

(6) $$c_{1}=\frac{1}{2}r_{st}x_{1}$$

(7) $$c_{2}=r_{st}\left(\frac{1}{2}x_{2}-\frac{1}{8}x_{1}^{2}\right)$$

(8) $$c_{3}=r_{st}\left(\frac{1}{2}x_{3}+\frac{1}{16}x_{1}^{3}-\frac{1}{4}x_{1}x_{2}\right)$$

(9) $$c_{4}=r_{st}\left(-\frac{1}{4}x_{1}x_{3}-\frac{1}{8}x_{2}^{2}+\frac{1}{2}x_{4}-\frac{5}{128}x_{1}^{4}+\frac{3}{16}x_{1}^{2}x_{2}\right)$$

and $x_{1},x_{2},x_{3},x_{4}$ can be found in [18] (Equations (10)–(13)).

$f_{D}$ and $f_{R}$ of the HREM can be expressed as

(10) $$\{\begin{array}{c} f_{D}=2c_{1}/\lambda \\ f_{R}=4c_{2}/\lambda \end{array}$$

Then, the deviation can be expressed as

(11) $$∆R_{st}\left(t\right)=R_{e}\left(t\right)-R_{st}\left(t\right)$$

The actual SAR echo signal can be expressed as

(12) $$s_{0}\left(\tau ,\;t\right)=A_{0}\cdot w_{r}\left(\tau -\frac{2R_{st}\left(t\right)}{c}\right)\cdot w_{a}\left(t\right)\cdot \exp\left(-j\frac{4\pi R_{st}\left(t\right)}{\lambda }\right)\cdot \exp\left\{jK_{r}{\left[\tau -\frac{2R_{st}\left(t\right)}{c}\right]}^{2}\right\}$$

where τ is the range time, $A_{0}$ is a complex constant, c is the speed of light, $K_{r}$ is the chirp rate, $w_{r}\left(\cdot \right)$ is the window in range, and $w_{a}\left(\cdot \right)$ is the window in azimuth. Ignoring $A_{0}$ and the windows, the above equation can be expressed as

(13) $$s_{0}\left(\tau ,\;t\right)\approx \exp\left(-j\frac{4\pi R_{e}\left(t\right)}{\lambda }\right)\cdot \exp\left\{jK_{r}{\left[\tau -\frac{2R_{e}\left(t\right)}{c}\right]}^{2}\right\}\times \exp\left\{j4\pi ∆R_{st}\left(t\right)\left[\frac{K_{r}\tau }{c}+\frac{1}{\lambda }\right]\right\}$$

By application of the principle of stationary phase (POSP) [27], the range Fourier transform of the above equation can be written as

(14) $$S_{0}\left(f_{\tau },\;t\right)={ℱ}_{\tau }\left\{\exp\left(-j\frac{4\pi R_{e}\left(t\right)}{\lambda }\right)\cdot \exp\left\{jK_{r}{\left[\tau -\frac{2R_{e}\left(t\right)}{c}\right]}^{2}\right\}\right\}\exp\left\{j4\pi ∆R_{\mathrm{st}}\left(t\right)\cdot \left(\frac{f}{c}+\frac{1}{\lambda }\right)\right\}$$

where ${ℱ}_{\tau }\left\{\cdot \right\}$ represents the range Fourier transform and $f_{\tau }$ is the range frequency. Then, the actual range history becomes an ideal hyperbola as described by Equation (1) after compensating

(15) $$\Phi_{1\mathrm{st}}=\exp\left\{j4\pi \cdot ∆R_{\mathrm{st}}\left(t\right)\cdot \left(\frac{f}{c}+\frac{1}{\lambda }\right)\right\}$$

in azimuth-time domain. This operation is just like the first-order motion compensation mentioned in [28], which can correct both phase and position. Even though Equation (15) is just valid for the reference target located in the center of imaged scene, the beam width is very small (about 0.287° in ST mode) and the correction is used on the whole imaged scene in this experiment.

After compensating the above derivation, a pure hyperbolic range history is forced, so that the classical imaging algorithms can be used without modification. But in the case of high-resolution, higher-order items, Equation (4) will play an important role in focusing. In this experiment, only third-order items were considered, and $c_{3}$ in Equation (4) can be obtained by fitting $R_{st}\left(t\right)$ and t. In general, $c_{3}\ll c_{2}$, so the quadratic stationary point calculated by the POSP can be approximated as cubic stationary point. Under this approximation, the azimuth quadratic stationary point can be written as

$$t=-\frac{f_{a}+f_{D}}{f_{R}}$$

Then, the cubic phase error can be compensated by

(16) $$\Phi_{\mathrm{cubic}}\approx \exp\left\{j\frac{4\pi c_{3}}{\lambda f_{R}^{3}}{\left(f_{a}+f_{D}\right)}^{3}\right\}$$

in range-Doppler domain, where $f_{a}$ is the Doppler frequency.

### 2.3. Two-Step Processing Algorithms

In ST mode, the Doppler bandwidth of a target located in the imaged scene can be expressed as $2X_{I}/\left(D_{az}X\right)$ [2], where $X_{I}$ is a synthetic aperture, $D_{az}$ is the antenna aperture in azimuth, and X is the length of the antenna footprint. Under the assumption that scene width is X in azimuth, the Doppler bandwidth of the whole scene is

(17) $$B_{a}=\frac{2X_{I}}{D_{az}X}+\frac{2}{D_{az}}=\frac{2X_{I}}{D_{az}X}+B_{as}$$

where $B_{as}$ is the Doppler bandwidth of stripmap. The $B_{a}$ in Equation (17) is usually much larger than the PRF. If we deal with the ST data in the way of the traditional stripmap SAR, then there would be a Doppler aliasing phenomenon in azimuth, which would introduce false targets in the focused image.

GF-3 uses the two-step processing approach—the de-ramped chirp scaling algorithm (DCSA)—to process the raw echo data. This algorithm involves two steps: the first step is prefiltering in azimuth, and the second step is dealing with the filtered data with the imaging approach of the standard stripmap SAR.

The first step implements an azimuth convolution between the raw data and a reference chirp signal $s_{ref}\left(t\right)$:

(18) $${\hat{s}}_{a}\left(t\right)=s_{a}\left(t\right){*}_{t}s_{ref}\left(t\right)=s_{a}\left(t\right){*}_{t}\exp\left(j\pi f_{Rref}t^{2}+i2\pi f_{Dref}t\right)$$

where t is azimuth time, $s_{a}\left(t\right)$ is azimuth raw data, ${*}_{t}$ is a convolution about t, ${\hat{s}}_{a}\left(t\right)$ is azimuth data after convolution, $f_{Rref}$ is the Doppler frequency modulation rate of the reference point (in the center of imaged scene), and $f_{Dref}$ is the Doppler centroid frequency of the reference point, and the value of $f_{Rref}$ and $f_{Dref}$ can be found in Table 2.

In a discrete domain, the above convolution can be converted to a fast Fourier transform (FFT) and complex multiplication shown in the below equation [4]

(19) $${\hat{s}}_{a}\left(m\cdot ∆t^{\prime \prime }\right)=\exp\left\{j\pi f_{Rref}{\left(m\cdot ∆t^{\prime \prime }\right)}^{2}\right\}\cdot FFT\left\{s_{a}\left(i\cdot ∆t^{\prime }\right)\cdot \exp\left[j2\pi f_{Dref}i\cdot ∆t^{\prime }+j\pi f_{Rref}{\left(i\cdot ∆t^{\prime }\right)}^{2}\right]\right\}$$

where $i=-\frac{N_{a}}{2},\cdots ,\frac{N_{a}}{2}$ and $N_{a}$ is the number of samples of raw data in azimuth, $m=-\frac{P}{2},\cdots ,\frac{P}{2}$ and P is the number of samples of prefiltered data in azimuth, $∆t^{\prime }$ is the sampling interval of raw data in azimuth, and $∆t^{\prime \prime }$ is the sampling interval of prefiltered data in azimuth.

In fact, the azimuth prefiltering is equivalent to reducing the signal duration while keeping the Doppler bandwidth unchanged. The result is that the azimuth sampling rate becomes higher than the Doppler bandwidth. Then, the problem of the Doppler aliasing phenomenon in azimuth would be resolved.

The second step is to use the classical CSA to deal with the prefiltered data. But in the process of azimuth filter and phase residual, the second-order term shown in Equation (20) should be added to eliminate the effects of the prefiltering.

(20) $$\Phi_{\mathrm{cor}}=\exp\left\{j\pi \frac{f_{a}^{2}}{f_{Rref}}\right\}$$

Then, the azimuth filter and phase residual can be realized by multiplying the azimuth signal with

(21) $$\Phi_{3}\left(\tau ,\;f\right)=\exp\left\{-j\frac{2\pi }{\lambda }c\tau {\left[1-{\left(\frac{\lambda f_{a}}{2V_{\left(r=\frac{\tau c}{2}\right)}}\right)}^{2}\right]}^{\frac{1}{2}}+j\theta_{∆}\left(f_{a},r\right)\right\}\times \Phi_{\mathrm{cor}}$$

and the above parameters can be found in [10].

### 2.4. Stop-And-Go Approximation

In the traditional airborne or low-orbit spaceborne SAR imaging algorithm, it is usually assumed that the radar does not move during the transmission of the pulse signal and the reception of the corresponding echo reflected from scatters (i.e., the stop-and-go approximation). Under this approximation, both the signal model and complexity of the imaging algorithm are greatly simplified. However, the approximation will introduce two adverse effects on image quality, especially in a high-resolution SAR system.

The first effect is that there will be a range-dependent azimuth shift in the focused image. In this staring experiment, between a pulse signal transmission and its reception, the satellite moves about 45 m. This shift can be compensated using a linear azimuth phase ramp $\Phi_{sg1}$ in range-Doppler domain after the range cell migration correction [22,24].

(22) $$\Phi_{sg1}\left(f_{a},\;\tau \right)=\exp\left\{j2\pi f_{a}\left(\tau -\tau_{s}\right)\right\}$$

where $f_{a}$ is the azimuth frequency vector, τ is the range time vector, and $\tau_{s}$ is the time delay of receiving the first range signal.

The second effect is that there will be a range-frequency-dependent azimuth shift in the echo signal. In the GF-3 case, the pulse duration is 45 μs, and the satellite moves about 30 cm. The range-time and chirp frequency have a one-to-one relationship, and then, the azimuth phase has a different slope in a different range frequency. Fortunately, this kind of shift is space invariant in azimuth, and it can be compensated using $\Phi_{sg2}$ in two-dimensional frequency domain.

(23) $$\Phi_{sg2}\left(f_{a},\;f_{\tau }\right)=\exp\left\{j2\pi f_{a}\frac{f_{\tau }}{K_{r}}\right\},f_{\tau }\in \left[-\frac{f_{s}}{2},\frac{f_{s}}{2}\right]$$

where $f_{\tau }$ is the range frequency vector, $K_{r}$ is chirp rate, and $f_{s}$ is range sampling frequency.

### 2.5. Antenna Pattern Modulation

GF-3 uses an active phased array antenna to achieve azimuth beam steering. However, the active phased array antenna has three drawbacks in practical application.

**Figure 3:**
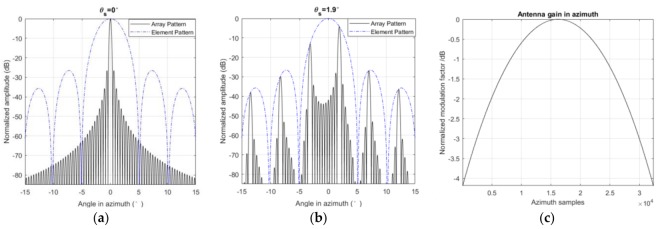
Antenna pattern of GF-3. (**a**) The antenna pattern when $\theta_{s}$ = 0°; (**b**) The antenna pattern when $\theta_{s}$ = 1.9°; (**c**) The normalized modulation factor of GF-3 antenna gain in azimuth in this experiment.

At first, the scanning range is limited by the distance between the two adjacent elements D, which is equivalent to the ratio of antenna aperture $D_{az}$, and the number of element $N_{az}$ in azimuth. If D is not small enough, grating lobes will appear and constrain the steering range. The position of the grating lobes $\theta_{g}$ can be described by Equation (24):

(24) $$\theta_{g}=\arcsin\left[\sin\left(\theta_{s}\right)\pm n\frac{\lambda }{D}\right]$$

where $\theta_{s}$ is the steering angle, λ is the carrier wavelength, and n is an arbitrary non-zero integer. Figure 3b shows the main lobe and the grating lobes between [−15°, 15°] when $\theta_{s}$ = 1.9°. There are six peaks in Figure 3b, and obviously, the highest peak is the main lobe and the others are the grating lobes. The interval between the two adjacent peaks is about 5.1°, which means that the maximum steering range is 5.1°. In operational SL or ST mode, GF-3 could steer from −1.9° to 1.9° in azimuth.

In addition, the antenna’s maximum power gain changes with the steering angle. Equation (25) shows the active phased array antenna pattern

(25) $$G=\sqrt{G_{0}}\cdot sinc\left(\frac{D}{\lambda }\pi \sin\left(\theta \right)\right)\cdot \sqrt{\frac{N_{el}}{N_{az}}}\cdot \left|\sum_{n=1}^{N_{az}}\exp\left\{j\frac{2\pi }{\lambda }nD\left[\sin\left(\theta \right)-\sin\left(\theta_{s}\right)\right]\right\}\right|$$

where G is the power gain of the array antenna, $G_{0}$ is the power gain of a single element, $N_{el}=64$ is the number of elements in elevation, θ is the look direction or azimuth antenna pattern angle, and $\theta_{s}$ is the steering angle. The envelop of array gain is modulated by a sinc(⋅) function and Figure 3a,b shows relationship between the steering angle and the antenna pattern. The influence of the element pattern is more obvious in TOPSAR [26]. In this experiment, 32,130 frames of data in azimuth were acquired, and the normalized modulation factor of every frame can be found in Figure 3c.

**Figure 4:**
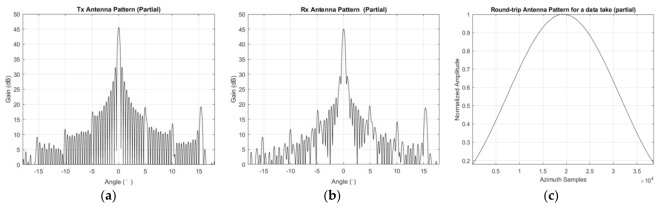
GF-3 azimuth antenna pattern of the data acquired in this experiment. (**a**) The antenna pattern within [−18°,18°] in transmit mode; (**b**) The antenna pattern within [−18°,18°] in receive mode; (**c**) The interpolated round-trip antenna pattern.

Finally, the antenna always illuminates the same scene on the ground in ST mode; therefore, the magnitude of images would be modulated by the antenna pattern. An intuitive phenomenon when the middle part of an image is brighter than the edges in azimuth Take the data acquired in this experiment as an example, we can get the antenna pattern in both transmit and receive mode with the help of auxiliary data, as shown in Figure 4a,b. According to the width of the imaged scene and distance of each pixel in azimuth, the beam width of the whole scene can be calculated. In this experiment, the beam width of the imaged scene is about 0.287° and the number of the azimuth samples in the final image is 38,588. In order to demodulate the antenna pattern, an interpolation operation is carried out on the round-trip antenna pattern, and the interpolated result can be found in Figure 4c. Then, the inverse of the curve shown in Figure 4c can demodulate the brightness of the focused image by every pixel at the image level.

### 2.6. Processing Flow

**Figure 5:**
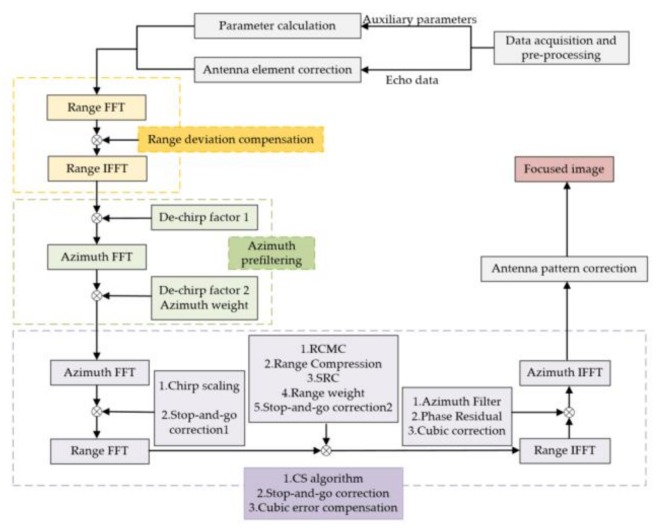
Data processing flow of GF-3 in ST mode.

In this subsection, a processing flow of GF-3 in ST mode is proposed and it can be found in Figure 5. After data acquisition and preprocessing, the echo data and auxiliary parameters are obtained. Then, the antenna element correction mentioned in Figure 3 is performed on the echo data and some parameters, such as the Doppler centroid frequency and the Doppler frequency modulation rate, are calculated based on auxiliary parameters. In azimuth-time domain, the first-order motion compensation forces the range history of the target into a perfect hyperbola, which is the foundation of the imaging algorithms. The azimuth prefiltering operation can suppress the Doppler aliasing phenomenon in azimuth. The next step is using the classical chirp scaling algorithm to deal with the prefiltered data. At the same time, the stop-and-go correction is carried out in range-Doppler and two-dimensional frequency domains. Before azimuth FFT, the cubic model error is corrected in range-Doppler domain. In order to suppress the sidelobes, Taylor windows are added in range and azimuth. The azimuth weight window was added in azimuth-time domain after prefiltering, and the range weight window was added in range-frequency domain. Finally, antenna modulation was compensated at the image level and a focused image was produced.

## 3. Experimental Results

In this section, an acquisition taken by GF-3 ST mode in Nanjing, China was used to verify the methodology mentioned in last section. Some parameters of the data are listed in Table 3. It should be noted that the steering angle range is [−1.78°,1.78°] in this ST experiment. According to Figure 1b, the theoretical azimuth resolution is about 44.7 cm. But the Doppler spectrum is weighted down by the antenna pattern and a Taylor window, and the actual azimuth resolution is about 54.5 cm.

### 3.1. Two-Step Algorithm Procession

**Figure 6:**
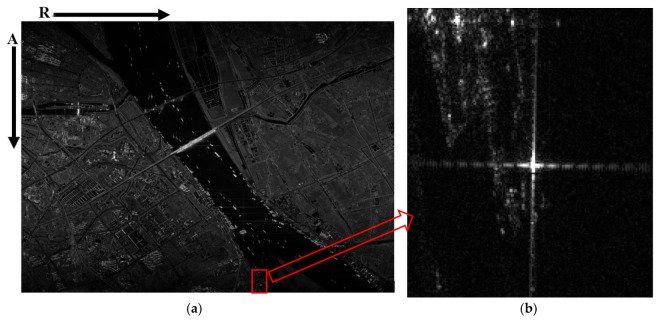
One primary image of GF-3 in ST mode. This image is processed by the two-step algorithm directly. (**a**) The whole image of one data take; (**b**) The local part of (**a**) which is a boat moored at the bank of the Yangtze River that we treat as a corner reflector.

The raw data is directly processed by the two-step processing algorithm and the primary image is shown in Figure 6a. We calculated some parameters of this image, and the result can be found in Table 4, where Na is the number of azimuth samples in the raw echo data, P is the number of azimuth samples after prefiltering, Ba_s is the Doppler bandwidth of the whole imaged scene, Ba_p is the Doppler bandwidth of one target within the imaged scene, and PRF_new is the azimuth sampling rate after prefiltering. Obviously, PRF_new is larger than the Doppler bandwidth (both Ba_s and Ba_p), and as a result, there will not be a Doppler aliasing phenomenon in azimuth. 

**Figure 7:**
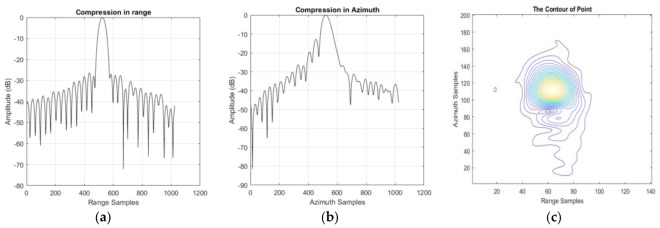
The compression result of the corner reflector processed by the two-step processing algorithm. (**a**) The compression result of the corner reflector in slant range; (**b**) The compression result of the corner reflector in azimuth; (**c**) The two-dimension contour in azimuth and slant range.

To analyze the quality of the focused image produced by our approach, a boat moored at the bank of the Yangtze River, which is shown in Figure 6b, is treated as a corner reflector; some results can be found in Table 5 and Figure 7. The overall improvement of the performance is related mainly to the peak sidelobe ratio (PSLR) and the integrated sidelobe ratio (ISLR), rather than to the resolution. So Taylor weight windows are added in both range (−24 dB) and azimuth (−19 dB). This operation can suppress the sidelobes effectively, but the main lobe will widen (i.e., lose resolution). The theoretical resolution is 0.625 m in range and 0.447 m in azimuth. After the weighting operation, the actual resolution is 0.731 m in range and 0.545 m in azimuth. It should be noted that the azimuth signal is not only weighted by a Taylor window, but also by the antenna pattern. Whereas calculating a precise antenna pattern window is very difficult because of the SNP, the actual azimuth resolution is the result of a simulation. The contour in Figure 7c is mussy.

### 3.2. Model Error Correction

**Figure 8:**
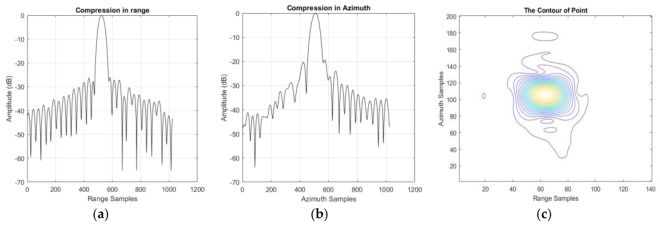
The compression result after model error correction. (**a**) The compression result of the corner reflector in slant range; (**b**) The compression result of the corner reflector in azimuth; (**c**) The two-dimension contour in azimuth and slant range.

As we can see from Figure 7b, asymmetric sidelobes, which are caused by a cubic phase error [5] (pp. 210–212) can be found in the compression results. At the same time, the PSLR and ISLR in azimuth are not perfect enough because of model error. After model error correction, the results can be found in Table 6 and Figure 8. Obviously, the performance in azimuth has been greatly improved: as azimuth resolution becomes higher, PSLR and ISLR become lower. Unfortunately, there still is a small amount of cubic phase redundancy in both range and azimuth. Meanwhile, the contour is still mussy.

### 3.3. Stop-And-Go Correction

**Figure 9:**
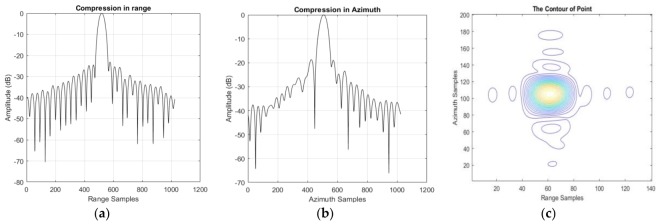
The compression result after stop-and-go correction. (**a**) The compression result of the corner reflector in slant range; (**b**) The compression result of the corner reflector in azimuth; (**c**) The two-dimension contour in azimuth and slant range.

The contour in Figure 8c is not tidy enough, and this might be caused by the stop-and-go approximation in the DCSA. After stop-and-go correction, the result can be found in Table 7 and Figure 9. Obviously, the compression result in both slant range and azimuth was better compared with results in the last subsection. The sidelobes can be found in the contour shown in Figure 9c. However, the performance is still not good enough and the sinc pulse seems a little noisy. The reason can be summarized as follows.

At first, in order to remove the deviation between the actual range history and the HREM, a novel formula shown in Equation (15) is compensated in azimuth-time domain. But Equation (15) can only remove the derivation of the reference target located in the middle of imaged scene. In reality, different targets have different range histories, and Equation (15) cannot remove the deviation of all targets located in the whole imaged scene. At the same time, we obtain the sensor’s real-time coordinates via the GPS receiver mounted on the satellite and then estimate orbit parameters. But the accuracy of the coordinates and the orbit parameters will influence the model error correction. If we use the coordinates provided by a dual-frequency GPS, a more accurate result might be obtained.

In addition, the boat is an integration of a set of scatters instead of an ideal point target, and other targets around the boat shown in Figure 6b will also influence the compression. 

Finally, as the pulse signal passes through atmosphere, refraction will occur. Then, the propagation path will be longer than the theoretical range history, and this might affect the parameters’ calculations and phase error compensation.

### 3.4. Antenna Pattern Demodulation

**Figure 10:**
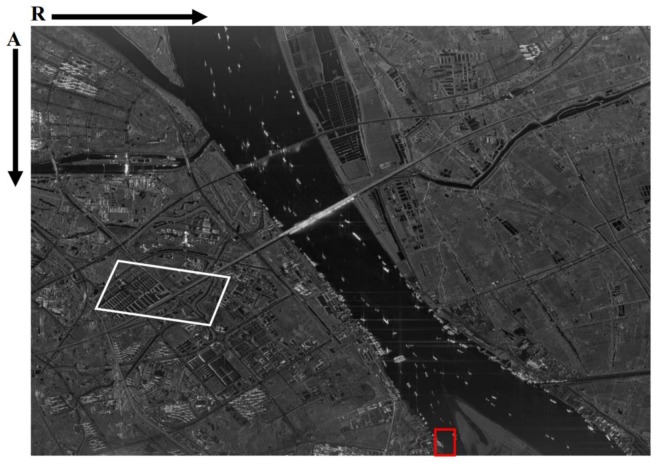
The final product of GF-3 in ST mode.

In ST mode, GF-3 always illuminates the same scene on the ground, so the brightness of images will be modulated by the antenna pattern, as Figure 6a shows. The inverse of the curve in Figure 4c can compensate for this modulation in the image level, and the result can be found in Figure 10. 

**Figure 11:**
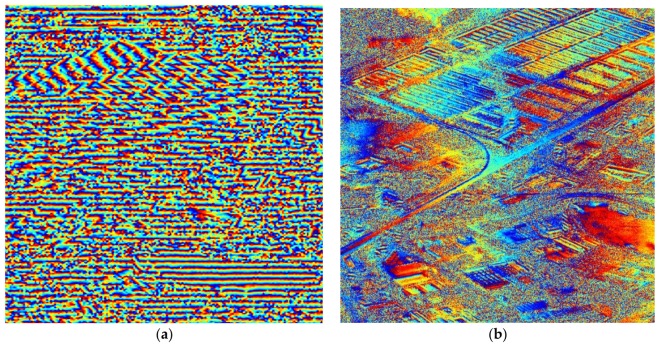
Some results of repeat-pass SAR interferometry experiment. (**a**) The interferogram of the spot marked with the white box in Figure 10; (**b**) The flattened interferogram of (**a**).

In order to verify the accuracy of the phase information, we conducted a repeat-pass SAR interferometry experiment based on the ST imagery above and a normal SL imagery on the same scene. The interferogram of the spot marked with white box in Figure 10 can be found in Figure 11a. Only when the data processing presented in this paper keeps the phase fidelity very well, can the ST focused image conjugate multiplied by the image obtained in regular SL mode effectively cancel the common backscatter to form an interferogram. More details of this interferogram experiment will be published in the future.

## 4. Discussion and Conclusions

This paper introduces an experiment regarding GF-3 ST mode. After data acquisition and pre-processing, some special aspects such as model error correction, stop-and-go correction, and antenna pattern demodulation are executed with a two-step processing algorithm, and the focused image shown in Figure 10 is produced. The interferogram shown in Figure 11 demonstrates that the data processing presented in this paper keeps the phase fidelity very well. This ST focusing experiment not only provides the highest-resolution image of GF-3, but also lays the foundation for the development of new higher-resolution Chinese SAR in the future.

Because the ST mode introduced in this paper is not a regular working mode of GF-3, users did not do a ST experiment in the calibration field at the commissioning phase, and we cannot get an image with ideal corner reflectors. The scene of the only attainable ST image shown in Figure 10 is very complicated, and it is difficult to find an ideal corner reflector. In order to obtain more precise results, another ST experiment needs to be applied at the calibration field.

ST is different from SL. The existing methods to remove SL cubic phase error in the range history is not suitable for ST. In this experiment, we calculated the cubic phase error for every pixel in azimuth, but this is time consuming. If the resolution becomes higher, the higher-order phase error will also influence focusing. Therefore, it is necessary to develop a new technology that can compensate cubic and high-order phase error efficiently.

In summary, there are still a lot of difficulties to be overcome in the future, and we will focus our efforts on solving these problems, especially the atmosphere’s influence on high-resolution images, at a later stage.

## Figures and Tables

**Table 1:** Some parameters of Gaofen-3 (GF-3)’s sliding spotlight (SL) and staring spotlight (ST) modes.

Observing Mode	Incidence Angle (°)	Az. Resolution (m)	Polarization	Bandwidth (MHz)
SL	20–50	1	HH	240
ST	20–50	0.45	HH	240

**Table 2:** Some parameters of acquisition geometry.

r (m)	$v_{e}$ (m/s)	$\cos\varphi_{e}$	$f_{D}$ (Hz)	$f_{R}$ (Hz/s)
934,633.16	7125.15	−0.000083	−21.21	−1956.82

**Table 3:** Some parameters of an image in ST mode.

Parameters	Value
Carrier Frequency	5.4 GHz
Pulsewidth	45 µs
Bandwidth	240 MHz
Sample Frequency	266.67 MHz
Pulse Repeat Frequency (PRF)	3742.15 Hz
Ve ^1^	7123.837734 m/s
Azimuth Steering Range	[−1.78°, 1.78°] ^2^
Step Steering Angle	0.01°
Look Angle	33.75°
Incidence Angle	38.51°
Synthetic Aperture Time	8.584 s
Range Window	Taylor (−24 dB)
Azimuth Window	Taylor (−19 dB)
Range Resolution	0.731 m
Azimuth Resolution	0.545 m

^1^ the equivalent velocity of the HREM, ^2^ even though GF-3 could steer from 1.9° to −1.9°, the steering range of the experimental image is from −1.78° to 1.78°.

**Table 4:** Some parameters of GF-3 ST mode data before and after de-chirp.

Na	P	Ba_p	Ba_s	PRF	PRF_new
32,130	38,588	16,809.30 Hz	19,379.69 Hz	3742.15 Hz	20,347.89 Hz

**Table 5:** The performance of compression just processed by two-step algorithm.

	Resolution (m)	PSLR (dB)	ISLR (dB)
Range	0.754	−26.343	−21.132
Azimuth	0.605	−12.776	−14.421

**Table 6:** The performance of compression after model error correction.

	Resolution (m)	PSLR (dB)	ISLR (dB)
Range	0.754	−26.138	−20.889
Azimuth	0.556	−19.445	−17.494

**Table 7:** The performance of compression after stop-and-go correction.

	Resolution (m)	PSLR (dB)	ISLR (dB)
Range	0.734	−23.085	−18.838
Azimuth	0.543	−18.679	−15.995
